# Monochrome Camera Conversion: Effect on Sensitivity for Multispectral Imaging (Ultraviolet, Visible, and Infrared)

**DOI:** 10.3390/jimaging8030054

**Published:** 2022-02-25

**Authors:** Jonathan Crowther

**Affiliations:** JMC Scientific Consulting Ltd., Egham TW20 8LL, UK; jonathan@jmcscientificconsulting.com

**Keywords:** UV, IR, monochrome, multispectral, calibration, validation, sensor

## Abstract

Conversion of standard cameras to enable them to capture images in the ultraviolet (UV) and infrared (IR) spectral regions has applications ranging from purely artistic to science and research. Taking the modification of the camera a step further and removing the color filter array (CFA) results in the formation of a monochrome camera. The spectral sensitivities of a range of cameras with different sensors which were converted to monochrome were measured and compared with standard multispectral camera conversions, with an emphasis on their behavior from the UV through to the IR regions.

## 1. Introduction

“Do you prefer to shoot in color or monochrome?” Few questions in photography can prompt discussion the way the one of whether color or monochrome is your preferred approach to imaging does. The first photographs were, of course, monochrome, but by the beginning of the 20th century, commercial processes for creating color photographs were starting to be developed [1]. In the early 1990s, with the first consumer-orientated digital camera, the Kodak DCS 100, a monochrome camera option appeared alongside a color version [2]. However as digital-camera manufacturers have continued to push the boundaries with sensor design, and increasing pixel count and dynamic range, outside of the machine vision and scientific camera areas, relatively few have created cameras with dedicated monochrome sensors [3,4]. Most consumer cameras use color sensors and include a way of creating monochrome images within the camera software, or the users can convert their color images to monochrome later, using a wide range of software packages. However, it should be remembered that the sensors themselves are originally monochrome, and the processes applied to them during manufacturing enable color images to be generated, typically through the use of a Bayer or color filter array (CFA) [5] or by relying on the depth of penetration of different wavelengths of light within the sensor [6]. If a monochrome image is the desired end result, the processing first to create a color image, and then to remove all that color information again, would seem to be overly complex, and could impact the quality of the final image [7]. These extra steps can be especially important factors if the photographer is interested in imaging in the regions beyond visible light, such as the ultraviolet (UV) and infrared (IR), and for multispectral imaging using band-pass optical filters or tightly defined light sources [8].

This article describes how the sensitivity of a range of monochrome converted digital cameras compares to both standard and multispectral converted (UV–Visible–IR) cameras. Sensitivity was assessed between 300 and 800 nm for a variety of test cameras with traditional sensor designs and backside illuminated (BSI) sensors. Enhanced UV sensitivity for the monochrome converted cameras was quantified by using a newly defined method, using narrow band-pass optical filters. Images in the UV and IR are also included here to demonstrate the observed sensitivity results. The relative contributions of the CFA and microlenses to the observed sensitivity are also discussed.

## 2. Materials and Methods

### 2.1. Camera Spectral Sensitivity Measurement

Camera spectral sensitivity was measured by using a custom-built device, using a method previously described in [9]. The sensitivity measurements are quantitative, in that they can be compared between different wavelengths and cameras, but they should not be confused with quantum efficiency values. The cameras discussed here are listed below:

- Standard unmodified Nikon d850.
- Nikon d850 UV–Vis–IR monochrome conversion with the CFA/microlenses and internal IR filter stack removed, and sensor cover glass replaced with a fused silica window.
- Standard unmodified Nikon d800.
- Nikon d800 UV–Vis–IR monochrome conversion with the CFA/microlenses and internal IR filter stack removed, and sensor cover glass replaced with a Schott WG280 window.
- Standard unmodified Sony A7III.
- Sony A7III multispectral conversion with the internal IR filter stack removed and the sensor cover glass replaced with a fused silica window.
- Standard unmodified Canon EOS 5DS R.
- Canon EOS 5DS R multispectral conversion with the internal IR filter stack removed and the sensor cover glass replaced with a Schott WG280 window.
- Canon EOS 5DS R UV–Vis–IR monochrome conversion with the CFA/microlenses and internal IR filter stack removed, and sensor cover glass replaced with a Schott WG280 window.

The multispectral and monochrome conversions were carried out by Llewellyn Data Processing LLC, Carlstadt, NJ, USA. The lens used for the sensor sensitivity tests was a Nikon Rayfact 105 mm f4.5 UV macro lens with appropriate lens mount adapter (the lens adapter did not contain any additional optics). Any variations for changes in ISO speed setting during the sensitivity measurements were corrected for during the data analysis. All images were saved as RAW files for analysis.

Error bars have not been included on the sensitivity graphs for sake of clarity; however, typically, the measured sensor-response values were within 3% of each other for multiple repeated measurements. However increased errors were observed for the multispectral cameras below 340 nm due to their relatively low sensitivity in that region (the unmodified cameras had no measurable sensitivity at 380 nm or below).

### 2.2. UVB/UVA Sensitivity Comparison between 313 and 365 nm 

Measurement of camera sensitivity at 313 and 365 nm (UVB and UVA regions) was carried out by using a Hamamatsu LC8 light source with a 200 W mercury xenon (HgXe) lamp, fitted with a custom made UV collimator. The lamp was run unfiltered and was left for at least 30 min to warm up before any imaging was carried out. A Labsphere 80% diffuse reflectance standard made from Spectralon^®^ was used as the test target. The test camera was fitted with a Rayfact 105 mm f4.5 UV macro lens with appropriate lens mount adapter (the lens adapters did not contain any additional optics). All images were captured at ISO400, and with a lens aperture of f8. Images were captured at a range of exposure times, using Edmund Optics 10 nm UV band-pass filters (313 and 365 nm OD4 hard-coated band-pass filters) mounted in a set of 52 mm filter adapter rings. Channel response of the cameras was plotted against exposure time.

### 2.3. UVA and UVB Images of Common Dandelion

Flower images at 365 and 313 nm were captured by using the monochrome and multispectral converted Canon EOS 5DS R cameras, and monochrome Nikon d850 camera used for the other experiments outlined in this article. The flower was a common dandelion (*Taraxacum officinale*) placed in a glass vase and imaged against white paper. A 20% Labsphere Spectralon^®^ diffuse reflectance standard was also imaged at the same settings as the flower to enable exposure to be compared at the two wavelengths between the cameras. Exposure times were based on the results of the sensitivity test. The light source was a Hamamatsu LC8 light source with a 200 W mercury xenon (HgXe) lamp, fitted with a custom made UV collimator. The lamp was run unfiltered and was left for at least 30 min to warm up before any imaging was carried out. A Rayfact 105 mm f4.5 UV macro lens with appropriate lens mount adapter (the lens adapter did not contain any additional optics). All images were captured at ISO400, and with a lens aperture of f16. Images were captured at a range of exposure times, using Edmund Optics 10 nm UV band-pass filters (313 and 365 nm OD4 hard coated filters) mounted in a set of 52 mm filter adapter rings. Images are shown in monochrome to enable visual comparison.

Filter-transmission spectra between 250 and 800 nm were measured by using an Ocean Insight DH-2000-BAL light source, Ocean Insight FX spectrometer using 600 µm extreme solarization fiber optic patch cords for the input and output fibers and 74-UV collimating lenses, mounted in an Ocean Insight RTL-T stand. The light source irradiance spectra were also measured by using the Ocean Insight FX spectrometer, using a 600 µm extreme solarization fiber optic patch cord and cosine corrector (measurement distance 50 cm from light source collimator).

### 2.4. IR Imaging Using Different Long-Pass Filters

IR sensitivity was compared for the Canon 5DS R monochrome and multispectral cameras, and the Nikon d800 and d850 monochrome cameras (the multispectral Sony A7III could not be tested as the test lens did not fit the available lens adapter). A Noct-Nikkor 58 mm f1.2 lens was used at f16, as this lens is reported to have good IR behavior [10]. Four Heliopan long-pass IR filters were used to assess different wavelength regions; Heliopan 715, 780, 830, and 1000. Images were taken at ISO400. A Labsphere Spectralon^®^ 60% diffuse reflectance standard was included with each image in order to check channel response at the different wavelengths. Lighting was overcast daylight (no direct sunlight), and all images were captured within a 15-min period. Exposure times were normalized for the Canon 5DS R multispectral camera with the different filters to ensure consistency (1/20 s for the 715 filter, 1/15 s for the 780 filter, 1/5 s for the 830 filter, and 4 s for the 1000 filter). These exposure times were then used for the other 3 test cameras.

### 2.5. Sensitivity Testing of Canon EOS 450d with Half the CFA/Microlens Array Removed

In addition to the cameras described above for tested for spectral sensitivity, the following was also assessed for sensitivity at 313 and 365 nm and in the 400–800 nm region; Canon EOS 450d with the internal IR filter stack and sensor cover glass removed, and the CFA/microlens array removed from half of the sensor, using the scraping method [11]. The sensitivity was measured in the three different regions of the sensor: (a) CFA and microlenses present, (b) CFA present but microlenses removed, and (c) CFA and microlenses removed (monochrome). The results were then used to calculate the transmission through the CFA and microlenses individually.

### 2.6. Image Analysis

Channel sensor response from the RAW files was carried out by using the approach outlined in [9], using RawDigger x64 Research Edition (LibRaw LLC, Potomac, MD, USA, version 1.2.23), and analyzed as RAW composite files (not RGB renders). All data were recorded, processed, and plotted, using Microsoft Excel 2010.

## 3. Results and Discussion

The camera conversions carried out for this research can be divided into two types. The first is multispectral conversion, where the internal IR filter stack is removed, along with the sensor cover glass (which is then replaced with a clear window of either fused silica or Schott WG280). The resulting camera can still capture color images, as the CFA is intact, but it can do so in wavelengths from UV to IR. The second is a monochrome conversion, which, similar to the multispectral conversion, removes the IR filter stack and sensor cover glass, but then it also removes the microlenses and CFA from the surface of the sensor. This produces a sensor which captures monochrome images from UV to IR, but lacks the color information.

There are two different sensor designs in the cameras being assessed. The Canon EOS 5DS R and Nikon d800 both have a conventional CMOS sensor design, while the Nikon d850 and Sony A7III both have backside illuminated (BSI) sensors. Cameras with both types of sensor were assessed to try to determine differences in behavior based on sensor design.

**Figure 1:**
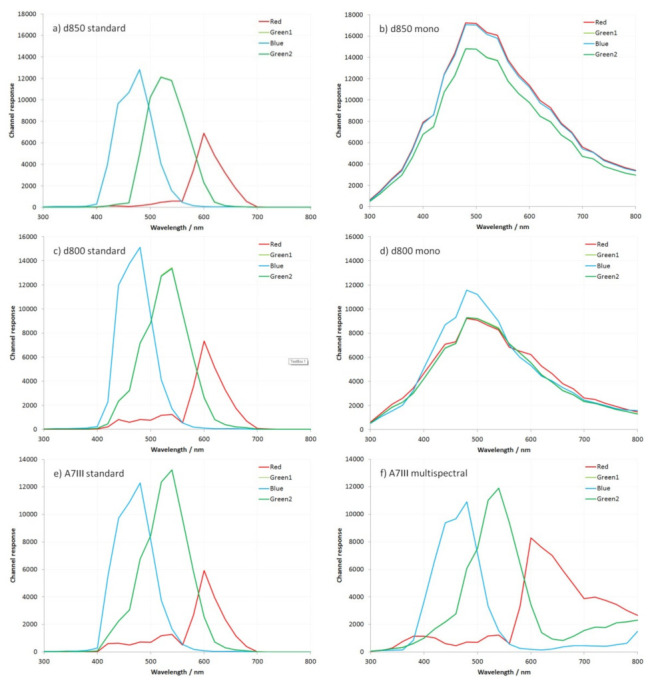
Measured sensor response curves between 300 and 800 nm for (**a**) standard Nikon d850, (**b**) monochrome-converted Nikon d850, (**c**) standard Nikon d800, (**d**) monochrome-converted Nikon d800, (**e**) standard Sony A7III, and (**f**) multispectral converted Sony A7III.

The camera spectral response was measured between 300 and 800 nm, in 20 nm intervals, using the method reported in [9]. Measured sensor responses for the test cameras for the Red, Green, Blue, and Green 2 color channels between 300 and 800 nm are given in Figure 1 (note the individual channel response data for the Canon EOS 5DS R cameras has been shown before in [9] and is not repeated here).

While with the manufacturers do not always release spectral response characteristics for their cameras, the shape of the red, green, and blue curves for the standard unmodified cameras in Figure 1, between 400 and 700 nm are comparable to those available in the literature [9,12,13,14,15,16]. The unmodified Nikon and Sony cameras show similar spectral responses for the different color channels. This is not surprising, as the both the Nikon d800 and Nikon d850 are reported to be using a Sony sensor [17,18]. The spectral-response curves for the two monochrome converted cameras shown in Figure 1 display the same profile shape, although they differ in the degree of sensitivity to the monochrome Canon EOS 5DS R discussed in [9]. The multispectral converted Sony A7III is similar to the multispectral Canon EOS 5DS R reported in [9]. With the monochrome conversions, sensitivity starts to become apparent below 300 nm, rising to a maximum at around 480 nm and then dropping again at longer wavelengths. The multispectral conversion has reduced sensitivity below 340 nm compared to the monochrome conversions. It should be noted that the monochrome converted cameras do not give an absolutely identical response in each of the color channels for all of the wavelengths; however, the differences between them are small, typically less than 0.3 stops. Possible reasons for why this is the case were discussed in [9].

**Figure 2:**
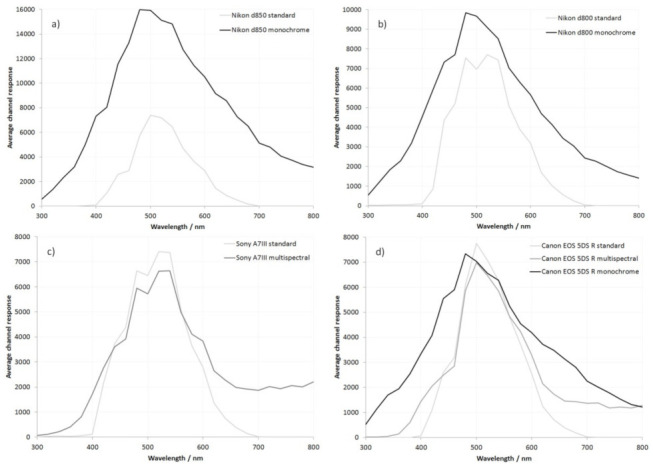
Averaged sensor channel response curves between 300 and 800 nm for (**a**) standard and monochrome Nikon d850; (**b**) standard and monochrome Nikon d800; (**c**) standard and multispectral Sony A7III; and (**d**) standard, monochrome, and multispectral Canon EOS 5DS R.

Average channel response as a function of wavelength for the different cameras, with and without modification, is shown in Figure 2. Note that different *y*-axis scales are used for the different test cameras.

As can be seen in the graphs in Figure 2, the standard unmodified versions of all the cameras had a similar average sensitivity as a function of wavelength. After conversion to multispectral, the Canon EOS 5DS R and Sony A7III cameras both showed a slight drop in average channel response at around 500 nm. The reason for this is not clear, although it could have to do with the nature of any anti-reflection coatings on the original internal IR filter stack and sensor cover glass. These would be optimized for light transmission in the visible region, so their replacement with a plain clear window with no anti-reflection coating could reduce the camera response, as less light would reach the sensor. The multispectral Sony A7III with its BSI sensor showed a greater IR response above 700 nm than the multispectral Canon EOS 5DS R.

Conversion to monochrome resulted in approximately the same maximum sensitivity for the Canon EOS 5DS R camera when compared to the standard unmodified camera and the multispectral converted camera. However, with the Nikon cameras, conversion to monochrome resulted in a large increase in sensitivity at all wavelengths, with the much larger improvement seen for the Nikon d850 with its BSI sensor. All three monochrome converted cameras showed a significant increase in the sensitivity in the UV region, and as the wavelength reduced to 300 nm, they were behaving similarly.

The principle differences between the standard and monochrome converted cameras are the absence of the internal IR filter stack, the sensor cover glass, and the microlenses/CFA. Removal of the CFA (which absorbs some of the incoming light) would be expected to increase sensitivity with a monochrome conversion; however, removal of the microlenses would reduce sensitivity, as light is no longer being collected from the non-sensitive parts of the sensor. Based on the results observed here, this would suggest that the removal of the microlenses is having a larger effect on the Canon EOS 5DS R than for the Nikon cameras. The Nikon d850 with its BSI sensor shows a much greater increase in maximum sensitivity compared to the Nikon d800, which has a conventionally designed CMOS sensor. In a BSI sensor, the sensitive part of the pixel is closer to the front surface of the sensor, rather than being inside a well [19]. Therefore, the removal of the microlenses would be expected to have less of a detrimental effect on sensor sensitivity for a BSI sensor than for the conventional sensor [20].

**Figure 3:**
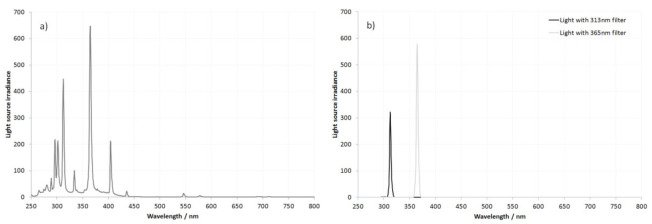
Irradiance spectra of the light source used for the exposure tests at 313 and 365 nm, (**a**) without filtering and (**b**) in combination with the 313 and 365 nm filters.

**Figure 4:**
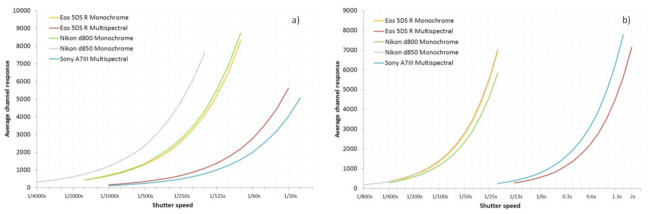
Average camera channel responses at (**a**) 365 nm and (**b**) 313 nm plotted as a function of exposure time (shutter speed).

One of the key benefits of these types of conversions is the ability to image beyond the visible part of the spectrum in the UV and IR. The use of UV for reflectance imaging has been reported for a wide range of applications, such as forensics [21,22,23], skin imaging [24,25,26,27,28,29,30,31,32], lepidopterology [33], botany [34], and historical conservation [35,36]. With a multispectral conversion which retains the CFA filter, the sensitivity in the UV drops rapidly, from 400 nm until around 350 nm, driven by absorption by the dyes in the CFA filter [37]. Conversion to monochrome removes the CFA filter and has been shown to significantly increase UV sensitivity [9]. The behavior of the test cameras in the UVB region at 313 nm and in the UVA at 365 nm was assessed by imaging an 80% diffuse reflectance Spectralon^®^ target under a range of different exposure settings. The light source was a 200 W mercury xenon lamp, which had sharp peaks at 313 and 365 nm, and band-pass filters were used the isolate the wavelengths of interest. The irradiance spectra of the light source, as well as wavelengths imaged by using the 313 and 365 nm filters, are given in Figure 3. The average camera channel responses at 365 and 313 nm were plotted as a function of exposure time. These are shown in Figure 4a,b.

As would be expected, with increasing exposure time at a given wavelength, the average channel response increases for all the cameras. By reading horizontally across the graphs in Figure 4, the relative sensitivity of the cameras at each wavelength can be determined. This is summarized in comparison to the monochrome-converted Canon EOS 5DS R camera in Table 1.

At 365 nm, the Nikon d800 monochrome camera is almost identical to the monochrome Canon EOS 5DS R, while the Nikon d850 monochrome camera is almost 1 stop faster. The multispectral cameras, with the CFA and microlenses still in place, are much slower at 365 nm than the monochrome cameras (2.3 stops slower for the Sony A7III and 1.7 to 2 stops slower for the Canon EOS 5DS R). At 313 nm, the difference between the multispectral and monochrome converted cameras becomes much more significant, with about 5 stops difference between them. Moreover, at 313 nm, the difference between the three different monochrome cameras essentially disappears, with at most 0.3 stops between them.

**Figure 5:**
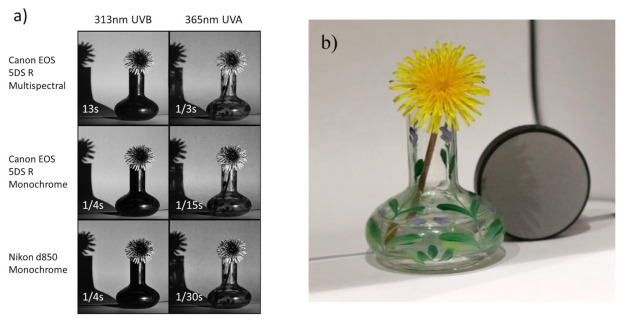
Images of a common dandelion (*Taraxacum officinale*) taken in (**a**) UVA (365 nm) and UVB (313 nm) with the test cameras and including exposure times; (**b**) visible light image of the subject and diffuse reflectance target.

Example images of a Common Dandelion (*Taraxacum officinale*) in a glass vase, using the 313 and 365 nm filters and three of the test cameras, and using the mercury xenon lamp, are given in Figure 5 (note the exposure times between the different cameras). All the UV images are shown as monochrome to enable comparison.

In the visible spectrum, the dandelion is a similar tone across the flower; however, under UV illumination, the central part of the dandelion, as with many flowers, looks dark as it strongly absorbs UV. Given its strong UV absorbance, dandelion-flower extract has been evaluated as a UV-protecting ingredient for use on skin [38], highlighting the useful role that UV photography can play in screening for potential candidates for UV-absorbing actives. The glass vase appears very different at 365 nm compared to 313 nm, going from transparent to opaque, as the glass strongly absorbs the short wavelength UVB light. In summary, conversion of the camera sensors to monochrome by removing the CFA resulted in a large increase in sensitivity in the UV region, and especially in the short wavelength UVB region. The monochrome converted Nikon d850 with its BSI sensor demonstrated increased sensitivity in the UVA region when compared with the other two test cameras; however, in the UVB region at 313 nm, this difference is no longer apparent.

**Figure 6:**
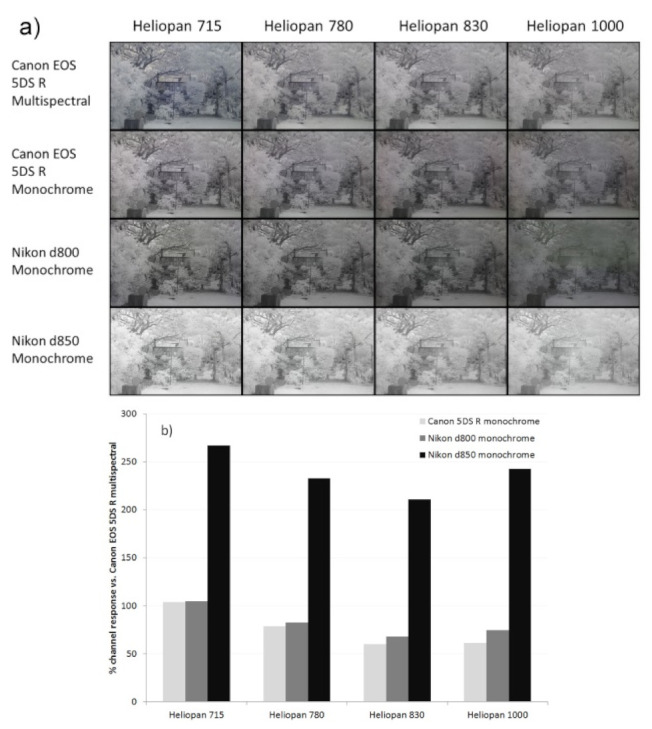
IR images taken with test cameras, using different IR filters: (**a**) direct image comparison; (**b**) average channel response of the diffuse reflectance target with the different cameras compared with the Canon EOS 5DS R multispectral camera for the different IR filters.

At the other end of the visible spectrum to the UV and above 700 nm is the IR region. Normal silicon based camera sensors are sensitive up to around 1100 nm [39], and IR imaging (i.e., above 700 nm) is often used by landscape photographers to darken skies and lighten foliage [40], and in forensics analysis of skin [41]. The method used to derive the spectral sensitivity curves in Figure 1 and Figure 2 can only be used up to 800 nm with the equipment available to the author. Imaging up to and beyond 800 nm was carried out by using a range of long-pass IR filters (Heliopan 715, 780, 830, and 1000) which let light through at increasingly longer wavelengths. A comparison of the test cameras while using each of these filters to capture a garden scene in daylight is shown in Figure 6.

In Figure 6a, the exposure times were set by using the Canon EOS 5DS R multispectral converted camera, and a Spectralon^®^ diffuse reflectance standard in the lower left part of each image. This was performed to ensure that the exposure was consistent with the different filters. The exposure times were then used to determine the sensitivity of each of the three monochrome cameras in relation to the multispectral Canon EOS 5DS R. Note that the Sony A7III multispectral camera could not be tested here, as the lens used for the test would not fit the adapter available for the camera. Visually, there are differences between the cameras in their response to IR. The monochrome Canon EOS 5DS R and Nikon d800 behave similarly to each other and show reduced sensitivity with the 780, 830, and 1000 filters compared to the multispectral Canon EOS 5DS R. The monochrome Nikon d850 has a much brighter image than the multispectral Canon EOS 5DS R with the all the filters, and this would be expected given the sensitivity data in the 700–800 nm region in Figure 1 and Figure 2. It should also be noted that the Noct-Nikkor 58 mm lens showed some evidence of “hot spotting” in the middle of the IR images, and this has been commented on before when the lens is used stopped down to f16 [10].

A quantification of the sensitivities of the cameras with each IR filter and a comparison of them against the multispectral Canon EOS 5DS R are shown in Figure 6b. As can be seen, the monochrome Canon EOS 5DS R and Nikon d800 both show a drop in response with the 780, 830, and 1000 filters. The monochrome Nikon d850 with its BSI sensor shows a much higher response than the other cameras. Above around 800 nm, the dyes used in the CFA become more and more transparent, blocking less and less of the incoming light. Therefore, the difference between the monochrome and multispectral cameras would be expected to reduce in the IR. However, with the monochrome conversion, the microlenses are being removed, which, as discussed above, would reduce sensitivity. It is the overall combined effect of these two factors which contributes to the lowering in sensitivity of the monochrome cameras with the standard CMOS sensors for the longer wavelength filters compared to the 715 filter. Interestingly, both monochrome Nikon cameras show a slight increase in sensitivity when using the longest wavelength 1000 filter when compared with the multispectral Canon EOS 5DS R. The reason for this is not clear; thus, it requires further investigation.

**Figure 7:**
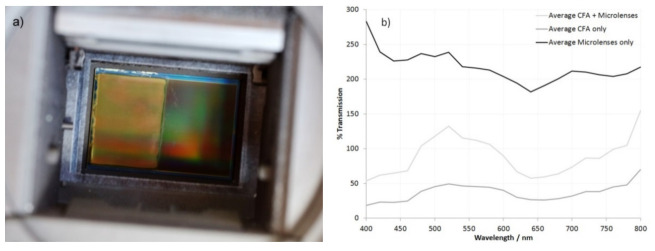

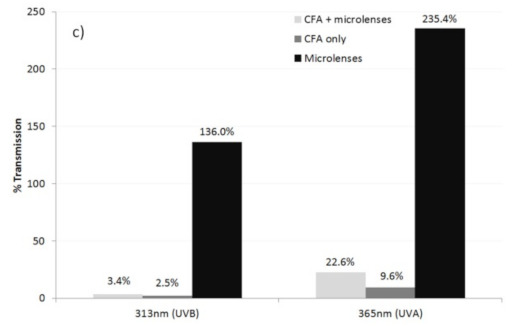
Sensor with half the CFA/microlens array removed; (**a**) appearance of the sensor; and percentage transmission between for the CFA+ microlenses, CFA only, and microlenses only, calculated from the average channel response; (**b**) 400 and 800 nm (**c**) at 313 and 365 nm.

As discussed above, the key difference between the multispectral and monochrome conversions was the CFA and microlenses on top of the sensor. During the monochrome conversions tested in this article so far, both the CFA and microlenses were removed, so it was not possible to individually assess the impact they had on sensitivity. Some people have carried out their own monochrome conversions by gently scraping the surface of the sensor with a wooden stick to remove the CFA and microlenses [11]. This is an extremely high-risk process which can easily damage the sensor, and, of course, it is very difficult to accurately control. While researching monochrome conversions, the author requested a sensor which had the CFA removed from half of a sensor, using this method. Essentially, half of the sensor was converted to monochrome, and the other half was left intact with the CFA and microlenses untouched. As a by-product of the scraping method, there was a region in the middle of the sensor which had the microlenses removed, but it retained the CFA. A photograph of the sensor showing the different regions is shown in Figure 7a. By using the approach outlined for Figure 1, and measuring the channel response at different wavelengths, it was possible to calculate how much light was absorbed by the CFA and microlenses separately. The average response for the different color channels is shown for the 400 to 800 nm region in Figure 7b. It should be noted that the transmission goes above 100% when the microlenses are present. This is because the microlenses are gathering light which would normally hit a non-sensitive part of the sensor, allowing it to be collected and imaged.

Unfortunately, the approach outlined above could not be used to generate data from 300 to 400 nm for this camera, as it was an older design with a relatively low maximum ISO speed setting. However, by testing with the 313 and 365 nm band-pass filters, using the method described for Figure 4, the relative sensitivity at these wavelengths for the different regions was measured, and it is shown in Figure 7c.

As can be seen from Figure 7b, across the visible and IR region, the microlenses act to increase the sensitivity compared to the CFA alone, which is what would be expected. Moreover, as discussed above, by 800 nm, the transmission of all three colors of the CFA is starting to increase, as the dyes used for the different color channels do not block the IR [42]. Although the measurements carried out here cannot go beyond 800 nm, the CFA essentially becomes equally transparent for red, green, and blue beyond approximately 800 nm. This explains the observed behavior with IR imaging while using the multispectral and monochrome cameras in Figure 6, where the multispectral camera (with the CFA and microlenses intact) showed a brighter IR image than the monochrome cameras with the same sensor type with the longer wavelength IR filters.

Below 400 nm, the CFA starts to block the UV (Figure 7c), with the UVB region showing more blocking than the UVA region. The microlenses are still working effectively to improve channel response in both the UVA and UVB, as shown by their transmission being above 100%. This was unexpected, as these are typically formed from polymeric material which could absorb the UV light [43], but it demonstrates that their presence can still be of benefit for UV photography. The improvement to the sensitivity offered by the microlenses was greater in the UVA region than the UVB region, presumably due to greater light absorption by the polymeric material at shorter wavelengths.

At this stage, it is only fair to highlight the limitations of this work described here. The sensor cover glasses were not the same on all the converted cameras. The monochrome converted Nikon d850 and multispectral Sony A7III had their sensor windows replaced with fused silica, while the monochrome converted Nikon d800 (and Canon EOS 5DS R conversions) had the sensor window replaced with Schott WG280. Given the spectral transmission characteristics of the Schott WG280 compared to those of fused silica, this would only be expected to make a difference at 280 nm or shorter wavelengths and should not influence the work presented here. The multispectral Canon EOS 5DS R and Sony A7III multispectral conversions were more extreme than a typical multispectral conversion, as they also had their sensor cover glass removed and replaced—most standard multispectral conversions leave the existing cover glass in place to minimize risk to the sensor during conversion. While it has not been possible to go into the details of internal camera filters in this article, the author has discovered that these sensor cover glasses can themselves be highly UV absorbing, especially in the UVB region. Therefore, the improvement offered by the monochrome conversions in the UV region could be even greater when compared with a multispectral camera, which retains the original sensor cover glass. Some commercial monochrome cameras exist, such as the Leica Monochrom and Phase One IQ4 Achromatic; however, the author was not able to obtain examples of these for comparative testing. Likewise, no commercially available monochrome machine-vision cameras were tested.

## 4. Conclusions

Although conversion to monochrome is a serious modification to make to a camera, removal of the CFA and microlens array can offer enhanced sensitivity to different wavelengths of light, especially in the UV region. This improvement will be of interest to both artistic and scientific photographers, and especially those interested in multispectral imaging. The behavior of the dyes in the CFA, the microlenses, and sensor design (either conventional CMOS or BSI) all play a part in the overall change in sensitivity offered by the monochrome conversion.

By using a controlled light source and band-pass optical filters, the behavior of a range of converted cameras was tested in the UVA and UVB regions, and quantification of their sensitivity was here reported. Moreover, it was possible to characterize the camera response in the IR region by using a set of long-pass filters with different cutoff wavelengths. Finally, the relative role of microlenses and CFA filter in determining the light transmission to the sensor, from the UV to the IR, was determined and quantified. While monochrome conversion of a Canon EOS 5DS R and Nikon d800, which both have conventional CMOS sensors, provided some improvement to sensitivity, it was the camera with the BSI sensor (the Nikon d850) which saw the largest increase in sensitivity to light as a result of the conversion.

Further work is planned to look more deeply into how the conversion to monochrome influences aspects of the image, such as resolution and tonality.

## Figures and Tables

**Table 1:** Relative sensitivity of test cameras at 313 and 365 nm compared to the monochrome converted Canon EOS 5DS R.

Test Camera	313 nm (UVB)	365 nm (UVA)
Canon EOS 5DS R multispectral	5.3 stops slower	1.7 to 2 stops slower
Nikon d850 monochrome	Same	0.7 to 1 stop faster
Nikon d800 monochrome	0.3 stops slower	0 to 0.3 stops slower
Sony A7III multispectral	4.7 to 5 stops slower	2.3 stops slower

## Data Availability

Data from this research are not available elsewhere. Please contact the author for more information, if required.
